# Safer not to know? Shaping liability law and policy to incentivize adoption of predictive AI technologies in the food system

**DOI:** 10.3389/frai.2023.1298604

**Published:** 2023-12-08

**Authors:** Carrie S. Alexander, Aaron Smith, Renata Ivanek

**Affiliations:** ^1^Socioeconomics and Ethics, Artificial Intelligence in the Food System (AIFS), University of California, Davis, Davis, CA, United States; ^2^Agricultural and Resource Economics, University of California, Davis, Davis, CA, United States; ^3^Department of Population Medicine and Diagnostic Sciences, Cornell University, Ithaca, NY, United States

**Keywords:** liability, knowledge, technology adoption, business, machine learning, regulation, AI ethics, economics

## Abstract

Governments, researchers, and developers emphasize creating “trustworthy AI,” defined as AI that prevents bias, ensures data privacy, and generates reliable results that perform as expected. However, in some cases problems arise not when AI is *not* trustworthy, technologically, but when it *is*. This article focuses on such problems in the food system. AI technologies facilitate the generation of masses of data that may illuminate existing food-safety and employee-safety risks. These systems may collect incidental data that could be used, or may be designed specifically, to assess and manage risks. The predictions and knowledge generated by these data and technologies may increase company liability and expense, and discourage adoption of these predictive technologies. Such problems may extend beyond the food system to other industries. Based on interviews and literature, this article discusses vulnerabilities to liability and obstacles to technology adoption that arise, arguing that “trustworthy AI” cannot be achieved through technology alone, but requires social, cultural, political, as well as technical cooperation. Implications for law and further research are also discussed.

## Introduction

AI technologies have become useful and highly relevant in the food system, for example, in leveraging federated learning to combat food fraud in food supply chains (Gavai et al., 2023) and adaptation for more efficient production and manufacturing processes (Misra et al., 2022; Konur et al., 2023). Many researchers, commentators, and government agencies are also interested in reining in the development and use of AI by companies who may exploit it for profit at the expense of public welfare. Much of the research and discussion focuses on AI needing to be developed and implemented in a manner that is “trustworthy” or “responsible” (Danks, 2019; Ryan, 2020; Braun et al., 2021; Mökander and Floridi, 2021; McGovern et al., 2022). These ethical considerations for the *use* of AI, while very important, make virtually no mention of the scenario presented here in which AI might be *avoided* at the public's expense. Decisions not to adopt an AI technology may at times be strategic, or may be due to a lack of sufficient resources, or both. There may also be many other complex variables in play as a company attempts to stay up to date with best practices while not over-extending itself with new capabilities it may not be able to handle. This article argues that “trustworthy AI” is a package deal that requires social, cultural, political, as well as technical cooperation.

Consider the following scenario for a firm in the food industry, such as a farmer, packer, processor, or retailer. Similar scenarios could arise outside the food system. The firm is deciding whether to adopt a new AI-based technology that will produce high-frequency granular information on the potential, expressed as risk measuring the probability and severity of adverse effects (Lowrance, 1976; Potter, 1996), for disease or pathogens to adversely affect food products or workers. Example technologies include a computer vision system designed to detect weeds for robotic weeding or ripe fruit for harvesting but which also could reveal fecal contamination that could indicate high risk of fecal pathogen presence; automatic detection of pathogens that may enter food products in processing plants; detection or prediction of contagious disease in food system workers or livestock. Upon receiving risk information, the firm may be able to mitigate risk or prevent an outbreak by taking potentially expensive actions such as ceasing production or recalling products. If the firm does not mitigate based on this information, then at a later date it may be held liable for damages. If it does not adopt the technology, and therefore never receives knowledge the technology might provide, then it may be less liable because it did not have the information required to prevent or mitigate the damage. The AI technology may also improve productivity. The firm will compare the mitigation and liability costs between the existing and new technology. If the new technology increases these costs by more than the value of any productivity gains from the new technology, then an economically rational firm will not adopt the technology.

What is of concern in this article is the recognition that legal experts may reasonably and pragmatically advise their business clients that taking on a powerful new algorithm that suddenly provides them with better data on when or where certain risks may occur may expose them to greater liability risk. This issue is similar to that noted by Wagner (1997) regarding knowledge and liability for toxicity. However, there are more potential costs than just those related to testing to acquire knowledge of risks and litigation regarding harms. An intermediate step, as this article argues, is that as a result of increased knowledge and the increased liability such knowledge may cause, companies may also need to make alterations to their products and processes to alleviate the risks. Such changes may be small in scope, or may involve significant and costly changes. This article will review findings from interviews, compare and contextualize these findings within law and economics literatures, and suggest possibilities for helping companies manage risks so that companies, workers, and the public can benefit from these new knowledge tools.

## Materials and methods

Confidential semi-structured interviews were conducted beginning in 2021 as part of on-going research funded by the AI Institute for Next Generation Food Systems (AIFS), an NSF/NIFA funded institute for AI food system technology research (Alexander et al., 2023). Interviews and one focus group have been conducted by C.A. To date, interviews have involved 66 researchers and stakeholders from all areas of the food system as part of two on-going projects, along with several surveys. The starting point and methodological foundations for this work came from bioethics research on transforming organizational culture from a culture of compliance to a culture of trustworthiness in the development and use of technologies that require and are accountable to the public trust (Yarborough et al., 2009). This research explores the perspectives of academic researchers in AI-related work as well as the views of stakeholders through a highly interdisciplinary mixed-methods approach including surveys, interviews, focus groups, quantitative analysis, and narrative analysis and inquiry (Walsham, 1995; Bøllingtoft, 2007; Yanow and Schwartz-Shea, 2009; Ybema et al., 2009; Hesse-Biber and Leavy, 2010; Schwartz-Shea and Yanow, 2011; Worline, 2012; James, 2017). The main purpose of these methods was/is to explore what “trustworthy” or “responsible” AI means to those creating or using it, and how food system stakeholders make decisions regarding whether AI technologies are trustworthy, reliable, or relevant enough to adopt them.

All efforts have been made to create an atmosphere of respect and safety where genuine engagement and reflection can flourish. Ethics review by UC Davis IRB determined that this research does not fall under human subjects research (IRB ID: 1709437-1, FWA No: 00004557, dated January 29, 2021, amended and reconfirmed, May 5, 2022). However, all possible measures have been taken to ensure the privacy and confidentiality of those being asked to participate. All AIFS researchers involved in the institute in 2021 were invited to participate, of whom 75% responded and agreed to be interviewed. Additional interviews were conducted through snowball sampling (Parker et al., 2019). Most interviews were conducted on Zoom, and most were recorded; all study participants consented to participation. All participants were advised that any recordings and transcripts would be kept confidential and anonymous in order to help participants feel safe responding candidly regarding sensitive ethical issues.

Due to the highly sensitive nature of the topic, demographic data that might compromise privacy were foreclosed from the outset, as a means of protecting identities of both participants and non-participants (Saunders et al., 2015). Therefore, the reporting of demographic statistics was restricted to stakeholder type. To date, interviewees described here include 45 researchers, staff, and board members representing more than 20 disciplines—many of whom work in multiple disciplines—who are affiliated with the funding institute. Of these, most are tenured, and a very small percentage are postdoctoral researchers or graduate students. In addition, participants include 21 stakeholders unaffiliated with the institute, including administrative, legal, or other government, professional, or advisory roles. A small number of stakeholders are indirectly related to food system research, but most come from within the food system, spanning all areas of food industry including agriculture, agricultural technology or “ag tech,” food packaging and distribution, and food recovery. The semi-structured interview guide for researcher interviews is available in the related article cited above (Alexander et al., 2023). All stakeholder interviewees were asked to describe the AI technologies they were familiar with or use and any challenges they have encountered or anticipate in adoption and use of these technologies. Follow-up questions arose spontaneously in a free-flowing conversation to gather in-depth information about the interviewee's perspectives. Interviews were analyzed inductively and key themes identified, as described by Alexander et al. (2023).

Scenarios were mentioned by a small number of food industry and researcher participants that revealed the problems with liability and adoption discussed in this article. Unrecorded follow-up meetings with researchers and legal professionals provided clarification and additional context. Based on these interviews and conversations, a preliminary codebook of emergent themes was developed by C.A. and then cross-referenced and contextualized within literature on economics and law to test and ground our findings. To ensure rigor and quality, themes were iteratively reviewed, discussed, refined, and agreed on by C.A., A.S., and R.I. This process draws on case study methods to “provide a richness and depth to the description and analysis of the micro events and larger social structures that constitute social life” (Orum et al., 1991) and “confirming…instances of theory” or “how social abstractions, such as concepts and theories, are played out at the level of experience” (McCormick, 1996). Due to the sensitivity of the subject matter and sample size, this study did not provide enough data to clearly indicate the precise balance of variables that tend to discourage or encourage adoption. However, the data and information we received indicate an urgent need for further research to gain more insights on the influence of legal concerns on AI technology adoption, especially in cases where technologies could support public and worker interests.

## Results

Findings from interviews suggest that a serious gap between liability law and AI technology adoption may exist or be worsening relative to available technologies for risk management. It is not so much that this gap is new, as that its relative size and impact are changing as data proliferate and AI technologies become available for food system sectors. This impact is changing in three ways: (1) as algorithms increase the possibility of early and precise identification and mapping of food system risks, the potential for mitigating them also increases; (2) the leap from knowing about risks to mitigating them is significant and depends on the probability and severity of adverse effects, cost of mitigation and the incentives of interested parties to mitigate; (3) the responsibility, or potential for being held legally liable, for *not mitigating* a known risk increases as known risks increase.

Adoption and use of AI technologies is thought to depend, at least in part, on trustworthiness—that is, producing the promised results or insights. However, the interviews reviewed here show that trustworthiness may actually disincentivize adoption of AI technologies. It may not be too much to say that in some cases, the better these algorithms work, the more of a risk they may pose for businesses, and the less likely a business may be to adopt them.

Such a scenario, at the very least, complicates assumptions about needing algorithms that are designed ethically or responsibly. It is not that such standards are not needed, but rather that without evaluating the legal and economic environments into which these technologies are released, standards of “trustworthiness” and reliability will be insufficient to support the adoption of algorithmic tools to identify and increase the capacity for mitigating risk in the public's interests. This raises questions about when and to whom the presumed “safety” benefits are provided by these technologies, which will be discussed below.

## Discussion

Much of the research on ethical development and use of AI has focused on issues related to “trustworthy” AI. Some literature supports the pursuit of these standards, while others critique them, but the focus is on the best practices to develop AI so that it prevents bias, ensures data privacy, and generates reliable results that perform as expected (Danks, 2019; Ryan, 2020; Braun et al., 2021; Mökander and Floridi, 2021; McGovern et al., 2022). Both EU and U.S. agencies have adopted these objectives in new and proposed frameworks for guiding the development of AI (European Commission, 2023; Raimondo et al., 2023). Furthermore, a new report by the FDA also demonstrates that the U.S. federal government is interested in adoption of new technologies that improve predictive capacity, reduce illnesses, and increase response times in a way that provides any necessary “confidentiality and proprietary interests” protections (U. S. Food Drug Administration, 2020). The broad goal is to use advanced AI technologies to create more visibility—that is, more knowledge—within the food system, making it safer and more resilient.

In theory, as AI or food regulation is put into place, or for any regulation that currently exists, those who violate the regulations or otherwise produce products or services that fall short of law and industry standards would be held liable. However, even when regulations are in place, they do not always produce the expected results. As Viscusi (2011) states, the “idealized world in which the tort liability system is supposed to produce efficient levels of safety is not how product liability law actually performs.” Viscusi argues that this disparity is due to large, unpredictable liability and insurance costs. He notes that these problems, in particular, tend to make product liability “a barrier to innovations that would reduce accidents” because of the many uncertainties in how courts will handle liability cases. This research is supported by other literature that indicates that legal systems are not functioning as intended or imagined (LoPucki and Weyrauch, 2000; Hopwood et al., 2014). While it is not yet clear whether or to what degree AI technologies would be managed under product liability law (Chagal-Feferkorn, 2019), AI is being used in the production of products that do fall under these laws.

Another study supports the conclusion that liability can influence adoption of technology. Dari-Mattiacci and Franzoni (2014) argue that “negligence rules tend to have a distorting impact on the technological path.” The examples that the authors analyze do not directly consider AI technologies, and relate only to those that would increase automation, not available knowledge. But this study suggests that liability laws can encourage adoption of technologies that reduce harm (to users, workers, or the public) when the “costs of care” for maintaining and using the technology, and the “costs of harm” under liability laws, are cumulatively lower than the costs of using an older technology. This framework has implications for the scenarios considered here, as will be shown in the sections that follow.

### Safer when?

The concern we highlight in this article is not that the technology creates more risk. It may or may not do that. Rather, because it provides more information or insights about risks that were already present, it potentially increases the responsibility and expense for mitigating those risks to bar against claims of negligence. The interviews suggest that adopting a technology that provides more knowledge of risk may invoke or trigger a responsibility for expensive risk mitigation (Gormley and Matsa, 2011). This potential increase in costs for risk mitigation may discourage companies from adopting the technology.

There is some debate about how AI will affect the economy (Furman and Seamans, 2019), but the scenario considered here is different than how technology, AI or not, is normally viewed within economics. In this case, it may not affect productivity directly, but rather increase knowledge regarding risks for workers and consumers. AI technologies that assess risks, for example, for the spread of disease among farm or food production workers or the spread of foodborne pathogens in the food supply, are not designed to make production more efficient, although some may improve quality (Shea, 1999). They are primarily designed to precisely map the timing and location of increased risks so that mitigation steps can be considered. In addition, these technologies may not offer direct cost-saving benefits that might compensate for the expense of adoption, as is the case, for instance, with labor-saving technologies that might reduce the number of employees needed.

These factors may create a situation for companies where it is “safer not to know,” and forego using the technology. In fact, the interviews suggest that, in cases where companies are in compliance with current legal standards, legal experts may at times advise their business clients not to adopt newer AI technologies. Then, should harm occur that results in a lawsuit, there will be less evidence to support charges of negligence (Connally, 2009). Seen from this perspective, it may be best, hypothetically, for a company not to investigate or seek more knowledge about risks than they have resources to mitigate. Particularly when these technologies are new enough that they have not become standard throughout an industry, being on the cutting edge of technology could work to the disadvantage of a company compared to its competitors.

Liability law follows various standards (Buzby et al., 2001). As Viscusi (2011) notes, “Under a negligence standard, firms will only be liable for product-related injury costs if the level of safety that they provide is below the legal standard.” Even under a no fault standard, and where workplace causation of a harm or illness may be presumed, such presumptions are rebuttable. For example, several states recently passed laws and governors issued executive orders classifying COVID-19 as an occupational disease, making at least some workers eligible for workers' compensation coverage, but even in such cases, many workers were denied coverage on the basis of causation (Duff, 2022). Liability cases involving compensation for employees have historically been minimal. As a recent legal commentary on workers' compensation observes, “The majority of early workers' compensation laws simply did not contemplate occupational diseases. Because these early statutes were largely a reaction to increasingly common industrial accidents, they were not well-suited to handle the often slower and less detectable onset of occupational diseases” (Moore, 2021). According to Moore, “the denial rate for occupational disease claims can be up to three times higher than the rate for injury claims.” This is due to the fact that employees are required to provide “causal proof that the employee's work materially contributed to the onset of the disease” (Moore, 2021). Particularly in such cases, where causation is defined as “increased risk” rather than “actual” or “proximate” cause (Duff, 2022), introducing a powerful AI tool that allows microbes to be predicted and mapped with new degrees of accuracy and certainty might have the potential to help companies take steps to mitigate risk, but these same tools may also greatly alter available evidence and long-standing practice and precedent in case law, such that adoption is discouraged.

The interviews suggest that companies loosely follow one of two broad approaches. Some companies may adopt as many new technologies as possible, actively digitizing data, and maximizing transparency, risk awareness, and risk mitigation. These companies may be larger and have the resources to take these steps. And they do so with the assumption that these technologies and methods will be the best means to protect themselves against reputational and financial damage (Seo et al., 2013). Such companies may see new digital and AI tools as methods to gain public trust in their brand, which, according to one food industry stakeholder, is a significant goal for most food producers. On the other hand, some companies may delay or choose not to digitize records and adopt these new technologies. This could be due to a lack of resources to fund a conversion from paper records and file cabinets to digital tools that could take years to complete and be very expensive to implement and maintain.

For these or other reasons, companies in many sectors may be hesitant or completely unable to commit to the use of tools that may, in addition to these conversion costs, leave them more exposed to liability. As industry norms shift and all companies throughout an industry begin to adopt certain practices that create expense, these expenses may be built into prices and passed on to consumers. But when competitors are not incurring these expenses or increasing potential exposure to liability, a company may choose to know more at its peril. If these liabilities disproportionately affect smaller companies, these trends may lead to smaller businesses closing with consolidation in the hands of larger and better-resourced companies.

### Safer for whom?

The question of whether it is “safer” for a company to adopt or not adopt AI technologies that increase knowledge of risk assumes that the companies are evaluating risk in terms of their own interests. Such a view may sell companies short and overlook genuine efforts to ensure quality and safety. Certainly, as indicated above with the concern for reputation and brand trust, public perception may be damaged if significant harm to public interests or wellbeing were to occur. While evaluating which methods to use or not use in terms of companies' costs certainly may provide benefits to workers and the economy, it also may implicitly prioritize a company's preservation over the interests of individual workers or consumers, or the public.

Limited liability has been noted for its controversial nature, as both a “birthright” of corporations (Rhee, 2010), and a “double-edged sword” that brings with it many economic benefits as well as problems (Simkovic, 2018). Scholars note that if a company adopts methods that may be in the public's interests but that ultimately lead to the company's bankruptcy or failure, then this may not only have a negative effect on that company's employees who will lose their jobs, but will also affect adjacent industries and people (Easterbrook and Fischel, 1985; Rhee, 2010; Simkovic, 2018). The employees of other companies may lose their jobs as investors and companies change course out of concern that their businesses may also come under scrutiny or be held liable in ways that make investment less profitable. Alternatively, surviving firms may become more profitable due to the elimination of competitors. This framing, particularly with regard to AI development and adoption, is sharply critiqued by opponents as grounded in determinism and supporting neoliberal policies that put profit above public welfare (Bourne, 2019; Greene et al., 2019). In other words, there is a tension that exists between those who argue that business interests and survival should be considered as a valid means of preventing broad worker and public harms extending beyond the failure of one business into the rest of the economy, and those who argue that business interests should never be prioritized above the immediate needs and wellbeing of workers of an individual business. This debate is unlikely to end in consensus and courts are left to determine when companies have prioritized their own profits and interests too much at the expense of its workers or public wellbeing.

This discussion highlights the positive externalities that food system AI technologies could bring to society (Lusk and McCluskey, 2018). Because the technology provides potential benefits not only to the company, but also to the public, the company cannot capture enough of the benefit to reap a sufficient return on its investment. The company then has an economic incentive to under-invest in the technology (Chaminade and Edquist, 2010; Hoffmann, 2016; Fuglie, 2018). Viewed in this way, the incentives of companies could be aligned with society by offering subsidies for adoption of the technology. However, especially in cases with unlikely but potentially severe outcomes such as serious illness or death of consumers or workers, adoption subsidies may never be large enough to offset the potential liability risk. This issue tests claims that AI can be developed and used in the interests of the public, because it is precisely such technologies that will be least likely to be adopted.

The changing standards in the use of AI and industry practices will likely change what courts may hold to be “foreseeable harms,” and this might help to encourage companies to adopt more knowledge tools to aid them in getting the best available advice and mitigating risks. As noted in the study by Dari-Mattiacci and Franzoni, courts may alter the “costs of harm” or negligence to encourage rather than discourage adoption of these technologies. As they state, “If the new technology reduces harm substantially, adoption should be encouraged: courts should relax the standard of the new technology and raise that of the old one” (Dari-Mattiacci and Franzoni, 2014). Specifically, they state that in the absence of financial incentives (both from cost savings and compliance and liability laws) to adopt a technology, companies may “disregard the effects of their technological choices on victims, [and] tend to under-adopt harm-reducing technologies” (Dari-Mattiacci and Franzoni, 2014). This being the case, Dari-Mattiacci and Franzoni recommend that for harm-reducing technologies, courts set the costs of liability for companies at a rate that encourages adoption. Also, following Wagner's (1997) proposed interventions, if a company did not use a predictive AI technology that might have shown increased risk of a harm and therefore provided an opportunity for the firm to prevent it, the company could be presumed to be below the standard of care, with liability costs assigned proportionally, or rebuttals of presumption of causation could be denied in favor of the plaintiff. This action would set the risk of not using newer predictive AI technologies above the risk of using them to better align the incentives of companies with those of society.

Likewise, in cases where AI technologies do not directly improve production, but rather provide new knowledge about risk, companies may be more or less likely to adopt these technologies depending on negligence rules adopted by courts. Shavell has shown that courts often attempt to set negligence standards on the assumption that companies have obtained optimal knowledge of risk. However, when courts find it difficult to assess what levels of knowledge about risk are optimal, courts fall back on setting negligence standards according to lower or “customary” levels of knowledge about risk (Shavell, 1992). As Supreme Court Justice Elena Kagan indicated in her well-publicized statement during oral arguments on Section 230 in February of 2023 (Gonzalez, 2023), the introduction of new AI technologies may leave courts unqualified to determine new technological capacities and implications. Courts may therefore set negligence standards at lower than optimal levels. Given that liability for worker illness is often difficult to prove (Moore, 2021), it is likely that liability costs in such cases will remain low, further disincentivizing adoption of AI technologies that provide higher than customary knowledge of risk.

### Moving forward

The changes in AI capability are happening rapidly, and adaptation from industries and courts may happen slowly. It may therefore be to the advantage of the public and society to create some type of temporary “on-ramp” for new technologies, for instance, in the first few years that a new use of AI is being tested and adopted. An “on-ramp” would assist companies who would like to begin experimenting with or using AI technologies to explore risks and strategies for mitigating them. Such an approach would also allow courts time to calibrate negligence rulings and make accurate determinations that will encourage companies to obtain optimal knowledge of risk and take appropriate levels of care (Shavell, 1992). An on-ramp may include instituting an “expiration date” for data and knowledge provided by AI—meaning that the predictions are only “usable” for a designated period of time, after which time they are self-destroyed or simply of no legal or other value—or providing resources for assisting smaller companies with digitizing older records where necessary in order to adopt newer digital record-keeping tools. AI technology developers might also build technologies to provide users with knowledge that has an inbuilt level of plausible deniability, such as through differential privacy algorithms that add noise to the data or output to obfuscate the true data (Qian et al., 2022). Additionally, new technologies might provide more than predictions and data, but also cost-effective recommendations for mitigating predicted or identified risks.

Also, there are calls for regulatory “sandboxes” to support the development of AI (Truby et al., 2022). However, these ideas target the possible risks of AI technologies to users, workers, and the public. The blind spot is considering the scenario of hesitance to adopt AI technologies when they could pose a benefit for users, consumers, workers, and the public, but create new costs for companies the beneficiaries are unwilling to pay for. AI regulatory sandboxes should also be extended to support companies in exploring the use of these technologies when the risks are not only to users, workers, and the public, but when risks and costs are also borne by the companies who adopt the technologies. In this case, a company might be permitted to expand what they know (or what is predicted) about workplace and product risks with some additional time, leniency, or support in choosing whether to adopt these technologies permanently and make the necessary modifications to facilities, processes, and training. Those willing to enter this experimental phase might benefit from additional access to resources for permanent implementation. This approach might have the effect not only of facilitating the initial adoption of risk mapping technologies, but may increase access to data and facilities for AI researchers who are working on developing these technologies. Such an on-ramp would support the development of reliable technologies that sustainably support worker, user/consumer, and public wellbeing. Finally, the “sandbox” could support development of legislation, for example, to explicitly recognize the level of certainty in the risk detection/prediction by an AI technology as a factor when evaluating liability claims.

Expecting companies to bear the full burden of adopting AI technologies may be unreasonable when those technologies provide potential harm reduction benefits to society but raise costs and liability risk for companies. Making a short-term or temporary “on-ramp” to facilitate adoption through adjustments to regulation, liability costs, and resources for transitioning to new technologies, may allow a smoother and earlier adoption of these technologies for the best interests of workers, consumers, and the public. This approach would have added benefits for smaller companies who may ultimately be squeezed out by larger companies as more advanced technologies and greater risk mitigation become the standard for reasonable care in food system industries.

## Data availability statement

The datasets presented in this article are not readily available because the identity and privacy of participants and confidentiality of interview material, transcripts, and recordings must be protected per agreement with participants. Deidentified data will be made available upon request with permission of participants. Requests to access the datasets should be directed to csalexander@ucdavis.edu.

## Ethics statement

The requirement of ethical approval was waived by University of California Davis Institutional Review Board for the studies involving humans because information was not collected about the participants but rather about business and organizational practices. The studies were conducted in accordance with the local legislation and institutional requirements. The Ethics Committee/institutional review board also waived the requirement of written informed consent for participation from the participants or the participants' legal guardians/next of kin because human subject research was not conducted. Nonetheless, consent was obtained from all participants.

## Author contributions

CA: Conceptualization, Data curation, Investigation, Methodology, Project administration, Writing – original draft, Writing – review & editing. AS: Conceptualization, Funding acquisition, Project administration, Supervision, Writing – review & editing. RI: Funding acquisition, Writing – review & editing.
